# Supplementary material for: Structural basis of the activation of c-MET receptor
Source: Nat Commun. 2021 Jul 1;12:4074. doi: 10.1038/s41467-021-24367-3 (PMC8249616; doi:10.1038/s41467-021-24367-3)
Supplement: Supplementary file 1 — Supplementary Information [file 41467_2021_24367_MOESM1_ESM.pdf]

Supplementary Information

Structural basis of the activation of c-MET receptor

Uchikawa et al.

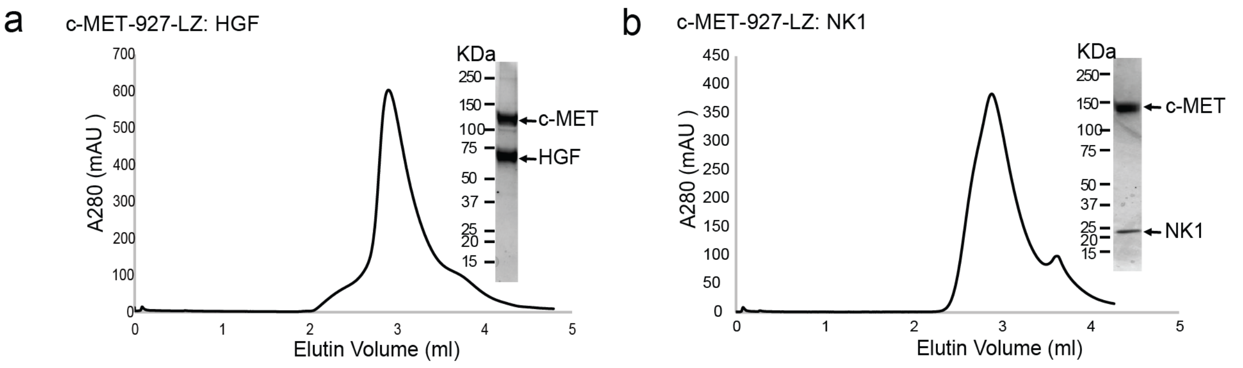

Supplementary Figure 1. Reconstitutions of c-MET/HGF and c-MET/NK1 complexes. The representative SEC profiles and SDS-PAGE analyses of the c-MET927-LZ/HGF **(a)** and c-MET927-LZ/NK1 **(b)** complexes, from N = 3 repeats. Heparin was added in both samples to promote the complex formation. C-MET/HGF or c-MET/NK1 were co-eluted in SEC, suggesting that the complexes were stably formed and remained intact during SEC.

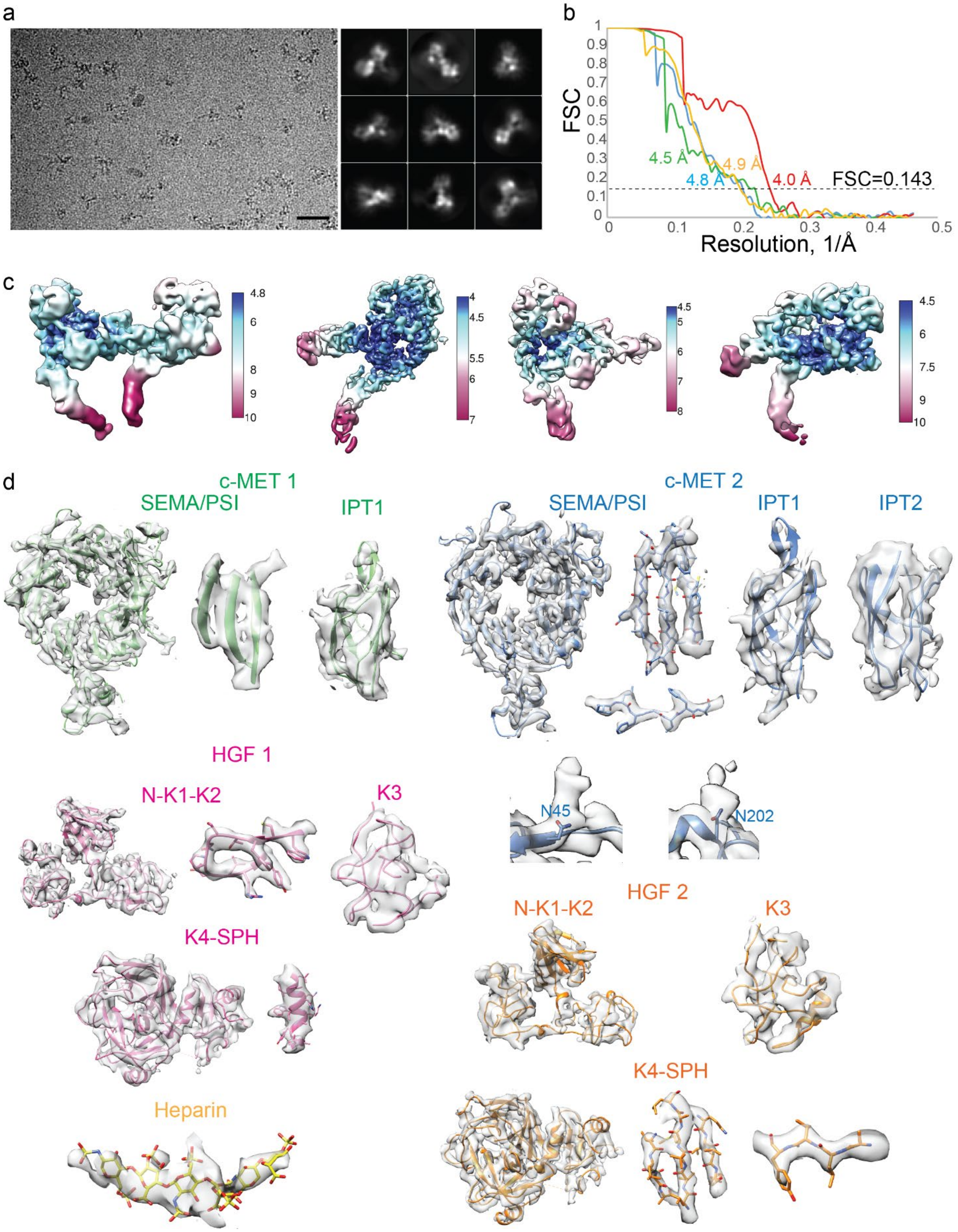

Supplementary Figure 2. Cryo-EM analysis of c-MET927-LZ/HGF complex

(**a**) Representative electron micrograph from total 8,100 micrographs and 2D class averages of c-MET927-LZ/HGF complex. Scale bar: 50 nm. (**b**) Gold-standard Fourier shell correlation (FSC) curve for the cryo-EM maps. Blue: cryo-EM map of holo-complex (2:2 c-MET/HGF complex). Red: cryo-EM map of sub-complex (1:2 c-MET/HGF complex). Green: cryo-EM map of c-MET I and HGF I obtained by focused refinement of holo-complex. Yellow: cryo-EM map of sub-complex after further 3D classification. A complete version of HGF II was resolved in this map. (**c**) Cryo-EM maps of c-MET/HGF complexes colored by local resolution. From left to right: cryo-EM map of holo-complex; cryo-EM map of sub-complex; cryo-EM map of c-MET I and HGF I obtained by focused refinement of holo-complex; cryo-EM map of sub-complex after further 3D classification, resolving the intact HGF II. (**d**) Representative cryo-EM densities of various parts of the c-MET/HGF complexes.

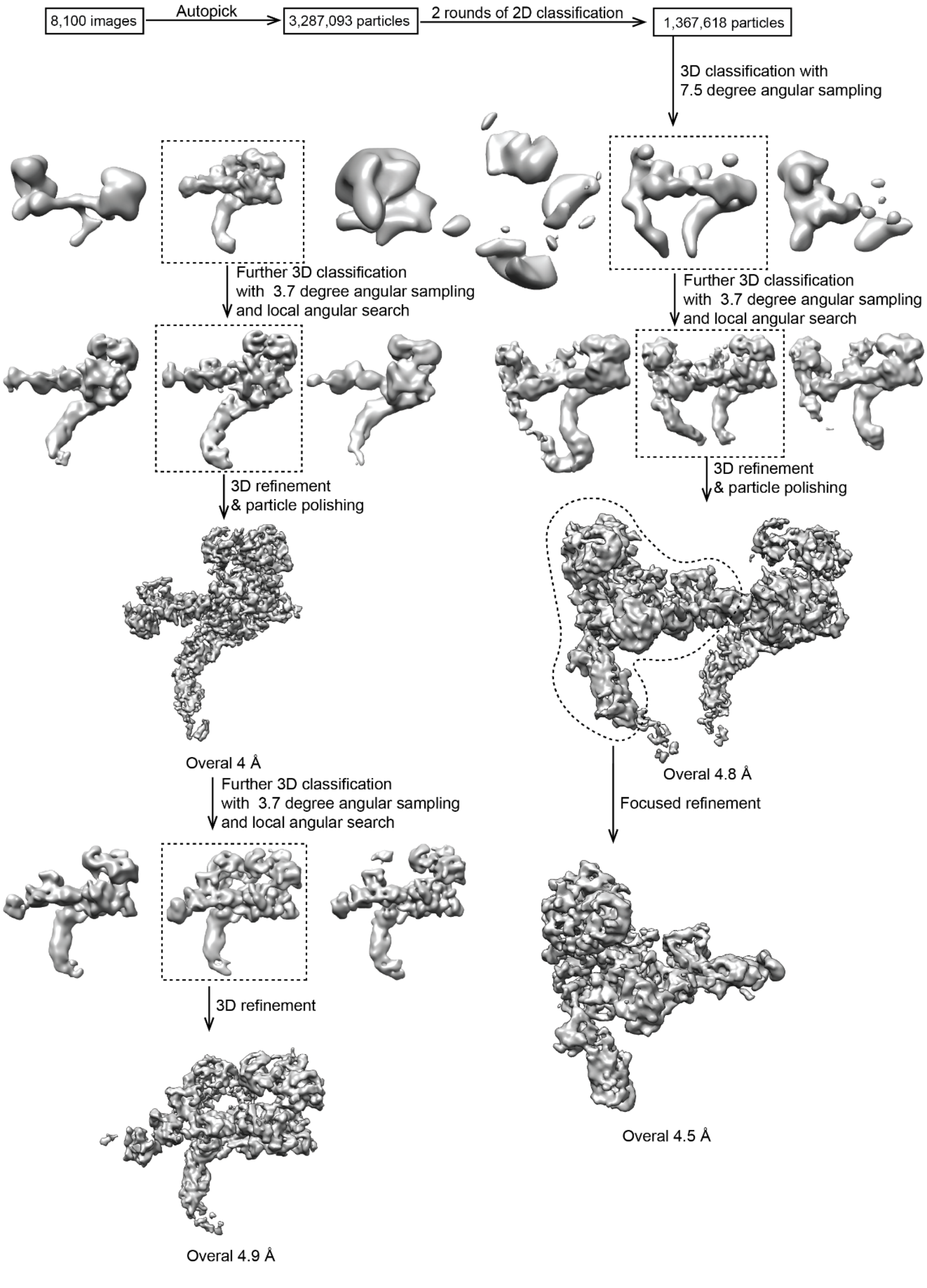

Supplementary Figure 3. Flowchart of cryo-EM data processing of c-MET/HGF dataset.

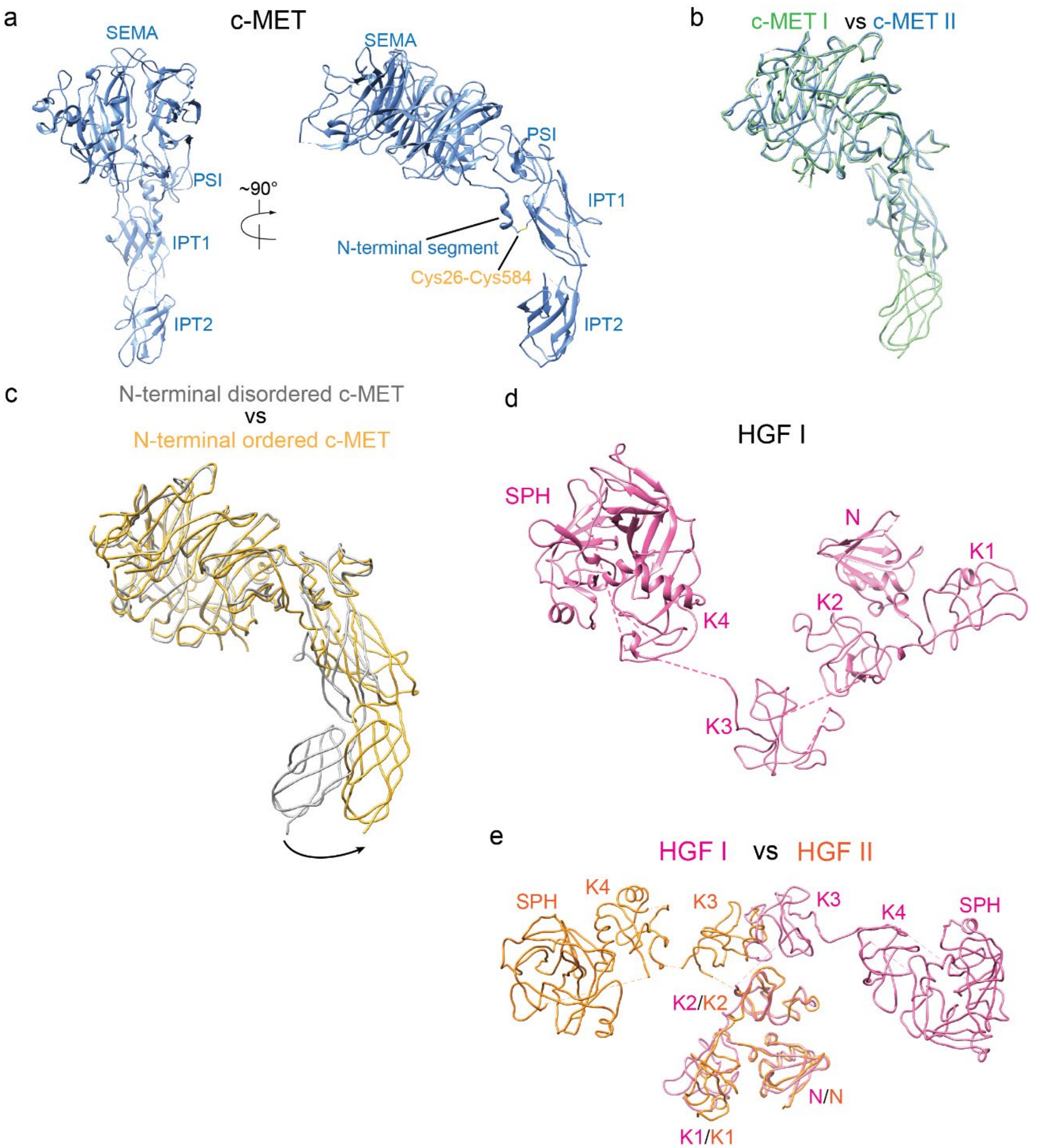

Supplementary Figure 4. The structure of c-MET extracellular domain and HGF.
(**a**) Overall structure of the c-MET extracellular region. The intramolecular disulfide bond (Cys26-Cys584) that links the N-terminal segment to the IPT1 domain is shown as yellow stick. (**b**) Superposition between c-MET I (green) and c-MET II (blue) by aligning the SEMA domain. (**c**) Superposition between N-terminal disordered c-MET from PDB ID: 2UZX[1] (grey) and N-terminal ordered c-MET from the cryo-EM structure of c-MET/HGF complex (yellow) by aligning the SEMA domain, revealing conformational change on the stalk region (indicated by an arrow). (**d**) Modular structure of the HGF. (**e**) Superposition between HGF I (pink) and HGF II (yellow) by aligning the two NK2 domains, showing the structural relocation of K3-K4-SPH segment in relative to NK2.

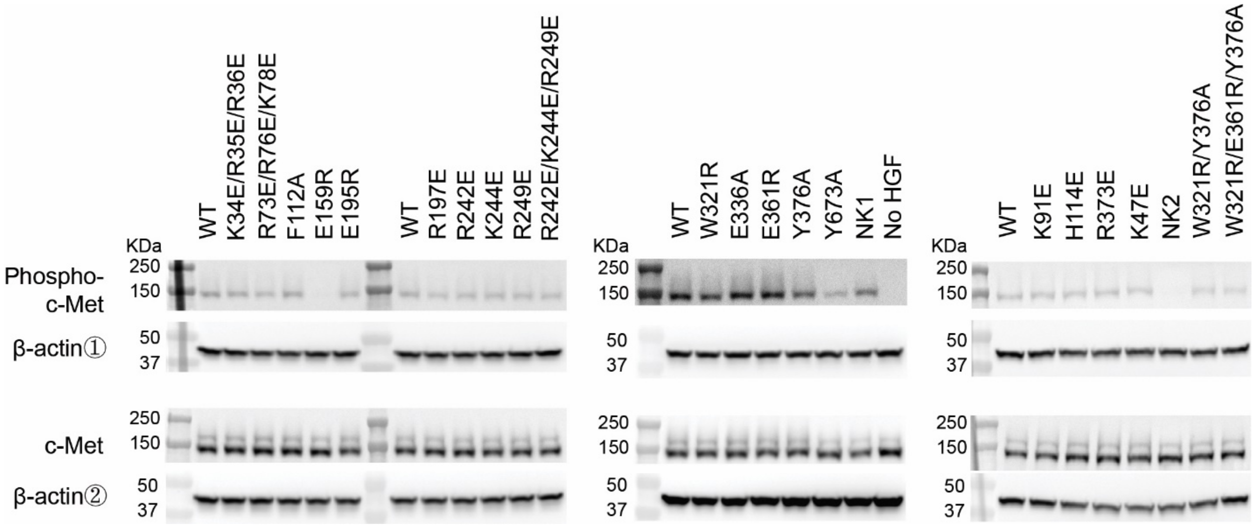

Supplementary Figure 5. HGF WT or mutants endow differential agonist activities. Representative western botting of Phospho-c-MET (Tyr1234/1235), c-MET, and loading control β-actin from N = 3 biological repeats. H1299 cells (~ 60% confluent in 6 well plate) was stimulated with 200 ng HGF (WT or mutant) for 10 min at 37°C. The agonist activity of each HGF variant was calculated following the formula: (Phospho-c-Met/β-actin①)/(c-MET/ β-actin②), and the presented agonist activity was normalized to HGF WT. Actin1 is the loading control for phospho-c-MET, while actin2 is the loading control for c-MET. Source data are provided as a Source Data file.

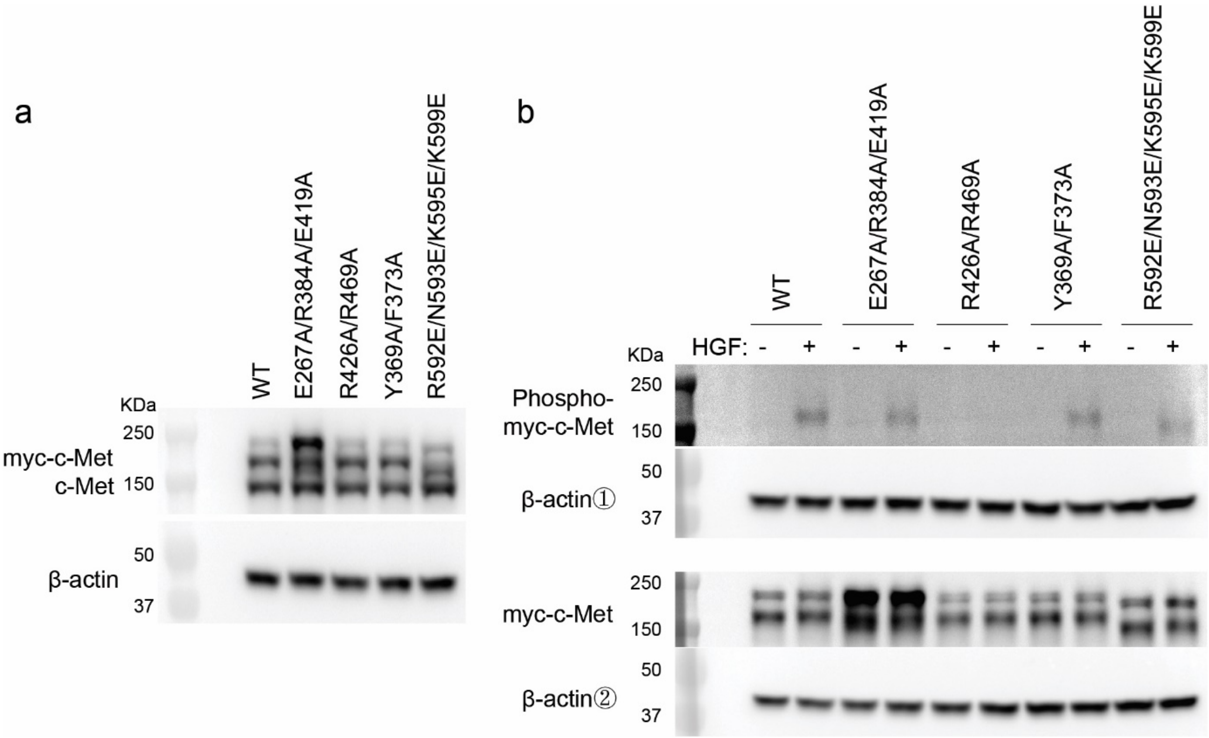

Supplementary Figure 6. HGF-induced auto-phosphorylation of c-MET in H1299 cells expressing full-length myc-tagged c-MET WT or mutants.
(**a**) H1299 cells stably express myc-c-MET (WT or mutants) were selected for functional assays. (**b**) Representative western blotting of Phospho-c-MET (Tyr1234/1235), c-MET, and loading control β-actin from N = 5 biological repeats. Endogenous c-MET was depleted by siRNA in H1299 cells stably expressing myc-c-MET, and then 200 ng HGF WT was applied for 10 min at 37°C to stimulate the phosphorylation of myc-c-MET. The phosphorylation capacity of each myc-c-MET WT or mutants was calculated following the formula: (Phospho-myc-c-MET/β-actin ①)/(myc-c-MET/ β-actin②). Here the basal auto-phosphorylation of myc-c-MET was subtracted for each variant and the presented agonist activity was normalized to myc-c-MET WT. Actin1 is the loading control for phospho-c-MET, while actin2 is the loading control for c-MET. Source data are provided as a Source Data file.

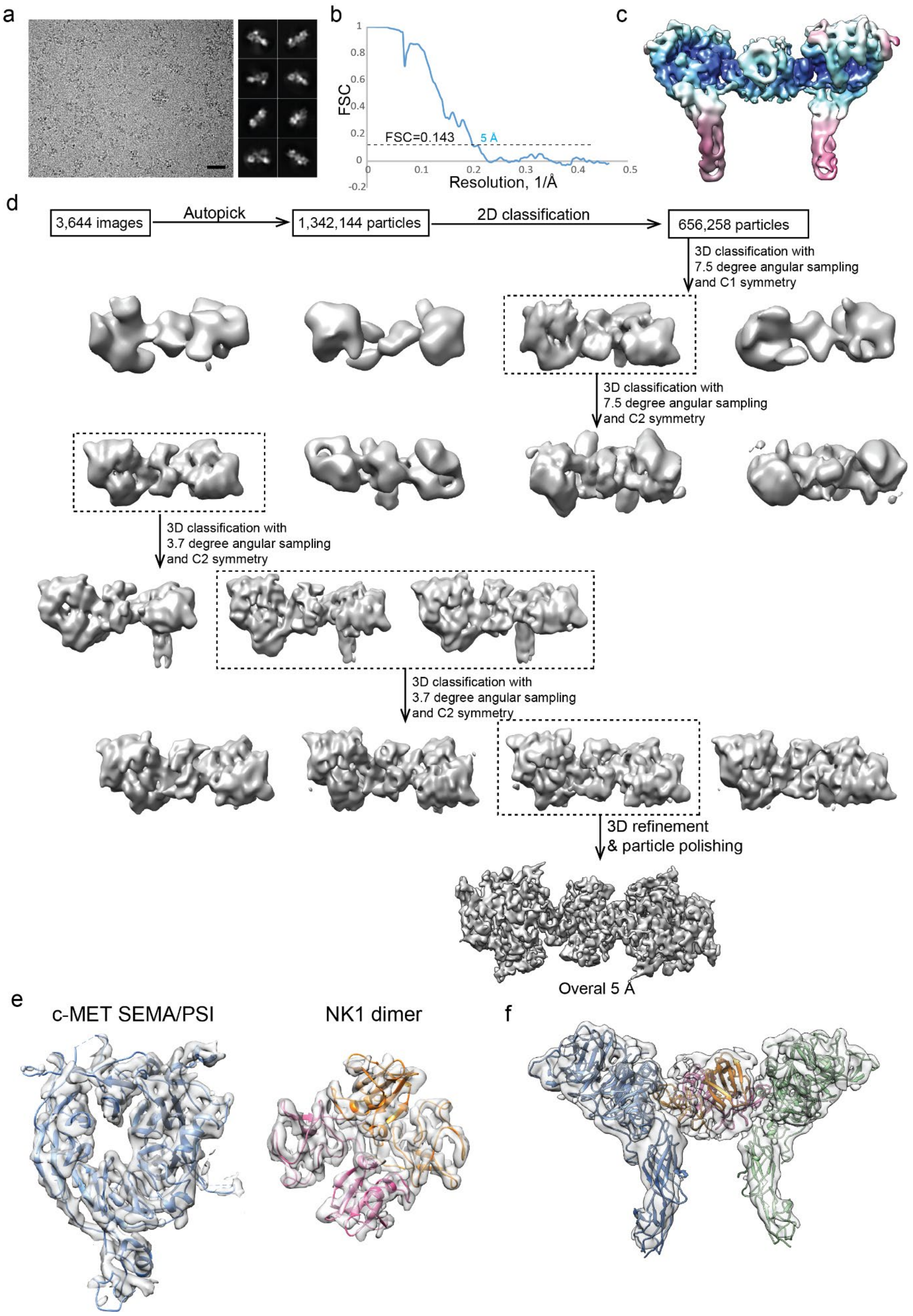

a
b
FSC
FSC=0.143
5 Å
Resolution, 1/Å
c
4.8
6
7
8
9
d
3,644 images
Autopick
1,342,144 particles
2D classification
656,258 particles
3D classification with
7.5 degree angular sampling
and C1 symmetry
3D classification with
7.5 degree angular sampling
and C2 symmetry
3D classification with
3.7 degree angular sampling
and C2 symmetry
3D classification with
3.7 degree angular sampling
and C2 symmetry
3D refinement
& particle polishing
Overal 5 Å
e
c-MET SEMA/PSI
NK1 dimer
f

Supplementary Figure 7. Cryo-EM analysis of c-MET927-LZ/NK1 complex.
(**a**) Representative electron micrograph from total 3,644 micrographs and 2D class averages of c-MET927-LZ/NK1 complex. Scale bar: 50 nm. (**b**) Gold-standard Fourier shell correlation (FSC) curve for the cryo-EM map of c-MET/NK1 complex. (**c**) Cryo-EM map of c-MET/NK1 complex colored by local resolution. (**d**) Flowchart of cryo-EM data processing of c-MET/NK1 dataset. (**e**) Cryo-EM densities of c-MET and NK1 dimer. (**f**) The rigid body fitting of the models of SEMA-PSI-IPT1-IPT2 domains of two c-MET and dimeric NK1 into the cryo-EM map that is low-pass filtered by local resolution.

Supplementary Table 1. Primers used in the study

Project	Oligo name	sequence
Stable cell line for functional test	cMetpLVXFW	CTATTTCCGGTGAATTCCTCGAGATGAAGGCCCCCGCTGTGCTT
	cMetpLVXRV	GGGGAGGGAGAGGGGCGGGATCCCCGTTCAAGTCTTCCTCGGAGAT
	pLVX_backbone_FW	GGATCCCGCCCCTCTCCCTC
	pLVX_backbone_RV	CTCGAGGAATTCACCGGAAATAGATCC
cmet and HGF for protein purification	cMetpEZTFW	TAATACGACTCACTATAGGCTAGCGCCACCATGAAGGCCCCCGCT
	cMetpEZT927RV	GAGTGGAAGCAAGCAATTTCTTCAGGTACCAGCAGCGGCCTGG
	HGF_FW	TAATACGACTCACTATAGGCTAGCATGTGGGTGACCAAACTCC
	HGF_RV	CCCCCGGGTCCTTGGAAGAGCACTTCGAGTGTCTCGCATGTTTTAAT TGCACAG
	HGF_NK1RV	GGTGGTGGTGGTGCTCGAGAACTTCTGAACACTGAGGAATGTCAC
cmet mutations for functional test	cmet_K267AR384AE419A_FW	CCTTTGGACCGTCAAGAAGTAAATAAAATTG
	cmet_K267AR384AE419A_RV	GAATATCGAACAGCCTTTACCACAGCTTTGC
	cmet_Y369A373A_FW	CACATGGCAGATCGATCCATTGGTTC
	cmet_Y369A373A_RV	CATGAGCACTGCTTTAATAGGACACTTCTG
	cmet_R426AR469A_FW	GGCCTGCAAAGCTGTGGT
	cmet_R426AR469A_RV	CTCATGTGAATTTTCTCCTGGACTC
	cmet_N382AF398A_FW	GGACATCTCACGGCGTTTTTGTT
	cmet_N382AF398A_RV	GATGAATATCGAACAGAGTTTACC
	cmet_K592EN593EK595EK599E_FW	GTCCCAGCCACATATGGTCAG
	cmet_K592EN593EK595EK599E_RV	CTTTAAGTGAGAGCACGATGAAT
HGF point mutations	343536_F	AGAGAATACAATTCATGAATTCAAAAAATCAG
	343536_R	TCCTCCCTTTGTCCCTCTGCATAG
	47_F	AAAATCAGCAGAGACTACCCTAATC
	47_R	TTGAATTCATGAATTGTATTTCTTC
	91_F	TGTTTTTGATGAGGCAAGAAAACAATG
	91_R	AAAGCCTTGCAAGTGAATG
	112_F	GAAAAAAGAAGCCGGCCATGAATTTGACCTC
	112_R	ACTCCACTTGACATGCTATTG
	114_F	AGAATTTGGCGAGGAATTTGACC
	114_R	TTTTTCACTCCACTTGAC
	159_F	GATACCACACCGGCACAGCTTTTTGCCTTC
	159_R	ATGGAACTCCAGGGCTGA
	195_F	AAGCAATCCACGGGTACGCTACGAAGTCTG

	195_R	GTGAAACACCAGGGTCCC
	197_F	TCCAGAGGTAGAGTACGAAGTCTGTGAC
	197_R	TTGCTTGTGAAACACCAG
	242_F	GACACCACACGAGCACAAATTCTTGCC
	242_R	TGATGATCCCAGCGCTGA
	244_F	ACACCGGCACGAGTTCTTGCCTG
	244_R	GGTGTCTGATGATCCCAG
	249_F	CTTGCCTGAAGAGTATCCCGACAAGGGC
	249_R	AATTTGTGCCGGTGTGGT
	242244249_F	TTGCCTGAAGAGTATCCCGACAAGGGCTTT
	242244249_R	GAACTCGTGCTCGTGTGGTGTCTGATGATC
	321_F	CAATACCATTCGGAATGGAATTCCATGTCAG
	321_R	ACAGTGCCCCTGTAGCCT
	336_F	GTATCCTCACCGGCATGACATGACTC
	336_R	TGAGAATCCCAACGCTGA
	373_F	TCCAAACATCGAGGTTGGCTACTGC
	373_R	TCAGTGGTAAAACACCAG
	361_F	AGATGGGTCTCGGTCACCCTGGTG
	361_R	GGATTTCGGCAGTAATTTTC
	376_F	CCGAGTTGGCGCCTGCTCCCAAA
	376R	ATGTTTGGATCAGTGGTAAAAC
	673_F	TGAGGGGGATGCCGGTGGCCCAC
	673_R	CATGGTCCTGATCCAATCTTTTC
	737678_F	GAATGAAGGACTTCCATTCACTTGCAAGGCTTTT
	737678_R	TCAGTACACTCATTAGCACATTGGTCTGCAGT

Supplementary References

1. Niemann, H. H. *et al.* Structure of the human receptor tyrosine kinase met in complex with the Listeria invasion protein InlB. *Cell* **130**, 235-246, doi:10.1016/j.cell.2007.05.037 (2007).
