# Supplementary material for: Structural basis of the activation of c-MET receptor
Source: Nat Commun. 2021 Jul 1;12:4074. doi: 10.1038/s41467-021-24367-3 (PMC8249616; doi:10.1038/s41467-021-24367-3)
Supplement: Supplementary file 3 — Reporting Summary [file 41467_2021_24367_MOESM3_ESM.pdf]

nature research

Corresponding author(s): Xiao-chen Bai; Xuewu Zhang

Last updated by author(s): Jun 1, 2021

Reporting Summary

Nature Research wishes to improve the reproducibility of the work that we publish. This form provides structure for consistency and transparency in reporting. For further information on Nature Research policies, see our Editorial Policies and the Editorial Policy Checklist.

Statistics

For all statistical analyses, confirm that the following items are present in the figure legend, table legend, main text, or Methods section.

n/a	Confirmed	
☐	☒	The exact sample size (n) for each experimental group/condition, given as a discrete number and unit of measurement
☐	☒	A statement on whether measurements were taken from distinct samples or whether the same sample was measured repeatedly
☐	☒	The statistical test(s) used AND whether they are one- or two-sided<br>*Only common tests should be described solely by name; describe more complex techniques in the Methods section.*
☒	☐	A description of all covariates tested
☒	☐	A description of any assumptions or corrections, such as tests of normality and adjustment for multiple comparisons
☐	☒	A full description of the statistical parameters including central tendency (e.g. means) or other basic estimates (e.g. regression coefficient) AND variation (e.g. standard deviation) or associated estimates of uncertainty (e.g. confidence intervals)
☐	☒	For null hypothesis testing, the test statistic (e.g. F, t, r) with confidence intervals, effect sizes, degrees of freedom and P value noted<br>*Give P values as exact values whenever suitable.*
☒	☐	For Bayesian analysis, information on the choice of priors and Markov chain Monte Carlo settings
☒	☐	For hierarchical and complex designs, identification of the appropriate level for tests and full reporting of outcomes
☒	☐	Estimates of effect sizes (e.g. Cohen's d, Pearson's r), indicating how they were calculated

Our web collection on statistics for biologists contains articles on many of the points above.

Software and code

Policy information about availability of computer code

Data collection	SerialEM 3.8
Data analysis	RELION3.1, MotionCorr2, GCTF1.18, Coot0.8.9, PHENIX1.17.1, Chimera 1.14, ImageJ 1.8.0_172, and Molprobity 4.5. All softwares are commercially or publicly available and described in the Methods section.

For manuscripts utilizing custom algorithms or software that are central to the research but not yet described in published literature, software must be made available to editors and reviewers. We strongly encourage code deposition in a community repository (e.g. GitHub). See the Nature Research guidelines for submitting code & software for further information.

Data

Policy information about availability of data

All manuscripts must include a data availability statement. This statement should provide the following information, where applicable:

- Accession codes, unique identifiers, or web links for publicly available datasets
- A list of figures that have associated raw data
- A description of any restrictions on data availability

We have now deposited all the 5 maps/models into PDB/EMDB database, including 2:2 holo-complex (PDB: 7MO7 [https://www.rcsb.org/structure/7MO7]; EMD-23919 [https://www.emdataresource.org/EMD-23919]), c-MET I/HGF I complex (PDB: 7MO8 [https://www.rcsb.org/structure/7MO8]; EMD-23920 [https://www.emdataresource.org/EMD-23920]), c-MET II/HGF I/HGF II (only contains K4 and SPH) (PDB: 7MO9 [https://www.rcsb.org/structure/7MO9]; EMD-23921 [https://www.emdataresource.org/EMD-23921]), c-MET/HGFI/intact HGFII (PDB: 7MOA [https://www.rcsb.org/structure/7MOA]; EMD-23922 [https://www.emdataresource.org/EMD-23922]) and c-MET/NK1 (7MOB [https://www.rcsb.org/structure/7MOB]; EMD-23923 [https://www.emdataresource.org/EMD-23923]). No restriction on data availability.

Field-specific reporting

Please select the one below that is the best fit for your research. If you are not sure, read the appropriate sections before making your selection.

☒ Life sciences ☐ Behavioural & social sciences ☐ Ecological, evolutionary & environmental sciences

For a reference copy of the document with all sections, see nature.com/documents/nr-reporting-summary-flat.pdf

Life sciences study design

All studies must disclose on these points even when the disclosure is negative.

Sample size	Sample sizes are given in the manuscript. All cell based c-MET autophosphorylation and pull-down binding assays were repeated at least three times, which is sufficient to derive error bars, p values and statistical significance.
Data exclusions	CryoEM data were processed in Relion, which excluded low-quality data to reach high-resolution using statistical methods. The exclusion criteria is pre-established as implemented in Relion, a common practice in cryo-EM.
Replication	All experimental findings were confirmed with at least three biological replicates as detailed in Methods or Figure Legends
Randomization	Each mutation of c-MET or HGF was combined with the wild type of the other protein in order to test the effect of the mutations on the HGF induced c-MET activation. No randomization is needed for this part. The structure determination procedure and other experiments followed standard procedures in the field that do not need randomization. For resolution estimation, all particles were randomly split into two groups.
Blinding	The investigators were blinded to group allocation during data collection and analysis.

Reporting for specific materials, systems and methods

We require information from authors about some types of materials, experimental systems and methods used in many studies. Here, indicate whether each material, system or method listed is relevant to your study. If you are not sure if a list item applies to your research, read the appropriate section before selecting a response.

Materials & experimental systems

n/a	Involved in the study
☐	☒ Antibodies
☐	☒ Eukaryotic cell lines
☒	☐ Palaeontology and archaeology
☒	☐ Animals and other organisms
☒	☐ Human research participants
☒	☐ Clinical data
☒	☐ Dual use research of concern

Methods

n/a	Involved in the study
☒	☐ ChIP-seq
☒	☐ Flow cytometry
☒	☐ MRI-based neuroimaging

Antibodies

Antibodies used	anti-c-MET antibody (8198S, Cell Signaling), anti-phosphor-c-MET antibody (Tyr1234/1235, 3077S, Cell Signaling), and anti-β-actin antibody (A1978, Sigma). The dilution used for each antibody is specified in the methods section of the manuscript.
Validation	C-Met antibody (Cell signaling 8198S) was validated to be rabbit species by goat-anti-rabbit IgG secondary antibody and its application in WB (see Extended Data Figure 5) detects two bands at around 145 kDa (mature c-Met) and 170kDa (immature c-Met). Phospho-c-MET antibody (Tyr1234/1235, Cell Signaling 3077S) was validated to be rabbit species by goat-anti-rabbit IgG secondary antibody and its application in WB (see Extended Data Figure 5) detects a band at around 145 kDa. Beta-actin antibody (Sigma A1978) was validated to be mouse species by goat-anti-mouse IgG secondary antibody and its application in WB (see Extended Data Figure 5) detects a band at around 43 kDa.

Eukaryotic cell lines

Policy information about cell lines

Cell line source(s)	Both Sf9 cells (11496015) and Freestyle 293-F cells (R79007) were purchased from Thermo Fisher. Naive H1299 cells were gifted from John Minna, and was originally obtained from the Hamon Cancer Collection (UT Southwestern Medical Center)
Authentication	We freshly purchased Sf9 (11496015) and 293-F (R79007) cell lines from Thermo Fisher and did not perform the additional authentication process. For the H1299 cells, cell line identity was confirmed by DNA fingerprinting (PowerPlex 1.2 Kit,

	Promega) and mycoplasma-free status was verified by PCR (e-Myco Kit, Boca Scientific, 25235).
Mycoplasma contamination	Negative for mycoplasma
Commonly misidentified lines (See ICLAC register)	No commonly misidentified cell lines were used.
